# Double-Blind, Placebo-Controlled, Randomized Study Comparing 0.0003**%** Calcitriol with 0.1**%** Tacrolimus Ointments for the Treatment of Endemic Pityriasis Alba

**DOI:** 10.1155/2012/303275

**Published:** 2012-04-22

**Authors:** Berenice Moreno-Cruz, Bertha Torres-Álvarez, Diana Hernández-Blanco, Juan Pablo Castanedo-Cazares

**Affiliations:** Dermatology Department, Hospital Central “Dr. Ignacio Morones Prieto”, Universidad Autónoma de San Luis Potosí, Avenida Carranza No. 2395, CP 78210, San Luis Potosi, Mexico

## Abstract

*Background*. Pityriasis alba (PA) is a frequent cause of consultation in tropical areas due to its chronic course, frequent relapses, and notorious hypopigmented lesions in pediatric dark skin populations. Currently, no treatment is widely accepted. *Objective*. To assess the efficacy of 0.0003% calcitriol and 0.1% tacrolimus ointments compared with placebo in the treatment of endemic PA. *Methods*. Twenty-eight children aged 3–17 years with 56 symmetrical lesions and phototype IV-V, were randomly assigned to receive the treatments on target lesions on the face. Improvement was evaluated at baseline and 8 weeks later clinically and by digital quantification of the affected area, colorimetry, and transepidermal water loss (TEWL). *Results*. Tacrolimus and calcitriol ointments induced a mean improvement of 68%, compared to 44% of placebo. We found an elevated TEWL in PA lesions. In the treated plaques, the reduction of the affected area was associated with improvement of pigmentation and TEWL. *Conclusions*. Calcitriol and tacrolimus induced similar repigmentation in endemic PA lesions. Melanogenic, anti-inflammatory, and barrier defect restoration properties of these drugs may explain these findings.

## 1. Introduction

Pityriasis alba (PA) is a benign inflammatory dermatosis affecting about 5% of pediatric population [1, 2]. Although it has a worldwide distribution, it is frequently seen in tropical areas of the world; therefore, it is more evident in individuals with darker complexion, although it is present in all skin types [1–5]. There are two types of PA, the endemic, affecting infants and children of low socioeconomic conditions in developing countries, and atopic-dermatitis-related PA which is associated with postinflammatory hypopigmentation [6]. This entity has received many synonyms (i.e., chronic impetigo, erythema streptogenes). It is characterized by hypopigmented, irregular plaques with well- to ill-defined borders, covered occasionally by fine scales; it affects mainly face, limbs, and sometimes thorax [1–3]. Its etiology is still unknown; although infectious mechanisms [3, 6], and nutrient deficiencies [1, 7, 8] have been implicated, a causal relationship has not been recognized. Excessive and unprotected sun exposure is considered an involved factor [1, 2, 8]. Another important risk factor is xerosis [1, 2, 4, 5]. Studies from stratum corneum of PA lesions have described defects in hygroscopicity and water-holding capacity detectable by water absorption-desorption test, this suggests that the condition is consequent to a dermatitic change and its hypopigmentation may be a postinflammatory mechanism [9].

Treatment includes topical application of humectants [1], corticosteroids [10], sunscreens [3], and antiseptics [10]. Recently, immunosuppressors (i.e., tacrolimus, pimecrolimus) have shown an excellent response in atopic related PA [11, 12]. However, there are no clinical trials confirming its usefulness in endemic PA. Calcitriol (1,25-dihydroxyvitamin D3) is an endogenous hormonally-active derivative of vitamin D. Receptors for calcitriol in keratinocytes modulate differentiation and inflammation [13–17]. It activates melanocytes, promotes melanin synthesis [18], and has immunomodulation properties [16, 17]. These features may be useful for the treatment of endemic PA. Adverse effects such as erythema and stinging have a low frequency [19], and its use, compared with other therapeutic options, would not imply a long-term carcinogenic risk or skin atrophy. Therefore, we compared the efficacy of the ointments of 0.1% tacrolimus, 0.0003% calcitriol, and the placebo for the treatment of endemic PA.

## 2. Material and Methods

### 2.1. Study Design

The investigation was an 8-week, randomized, double-blind, split-face placebo-controlled trial. The study was conducted at the Dermatology department of the Hospital Central of San Luis Potosí, México (latitude 22°09′ north, altitude 1877 meters). Informed consent was obtained from patients and parents before entering the study, which was approved by the local ethical committee. The trial is registered at the US National Institutes of Health Clinical Trial Register, with the number NCT01388517.

### 2.2. Patients

Patients from both genders, between 2 and 18 years old, attending our outpatient clinic, were included. Subjects affected by symmetrical lesions of PA on the face, between 2 and 6 cm^2^were selected. Hypopigmented areas on the face were initially evaluated, and target lesions were identified for follow-up. Patients with other dermatosis, including atopic dermatitis, and those who had used any systemic or topical medication during the past 6 weeks were excluded. The demographic data of the patients are shown in Table 1. 

### 2.3. Treatment

Topicals were set in similar containers and patients were randomly assigned in a double-blind manner to receive, 0.0003% calcitriol (Galderma, France), 0.1% tacrolimus (Astellas Pharma, USA) or placebo (petrolatum, Sigma Aldrich, USA) on target lesions. All subjects were instructed to apply the treatment twice daily. Sunscreen was not indicated, but the use of any type of cleanser or cosmetic product on lesions was not allowed. All adverse effects were recorded.

### 2.4. Assessments

Patients were examined at 2, 4, 6 and 8 weeks. The primary outcome measure was the reduction of the affected area. Clinical improvement was appraised by means of digital photographic registration (frontal, right, and left views). Images were analyzed using the software ImageJ v1.4, to quantify the dimension of target plaques. As previously described, lesion was given a 0% score at baseline, and a second percentage value was obtained at the end of study [20]. An independent observer performed a Physician Global Assessment (PGA), which was scored as poor (0–25%), mild (26–50%), good (51–75%), and excellent (>75%).

Pigmentary changes were objectively evaluated using a colorimeter (Chromameter CR-300, Minolta, Osaka, Japan). Coloration changes were examined by means of the L* (luminosity), where 100 value is full white, and 0 total black; and the a* (erythema) axis from 0 to 50. Improvement was assessed by obtaining the L* axis difference (L*Δ) between the targeted lesional and perilesional areas (L*Δ = lesional − perilesional). Skin that was at least 1 cm away from apparent lesion was considered perilesional. This was performed for each visit during the study.

A secondary outcome measure included the evaluation of the integrity of the cutaneous barrier, at baseline and its modification by treatments. Transepidermal water loss (TEWL) was measured by an evaporimeter (Dermalab, Cortex Technology, Denmark). Water loss was recorded on each plaque in g/m^2^/h. Prior to measurement, volunteers were rested for at least 20 min at 19–21°C in a relative humidity environment of 40–45%. Corneal integrity changes were appraised by the TEWL difference between the targeted lesional and perilesional areas (TEWLΔ = lesional − perilesional).

### 2.5. Statistical Analysis

We calculated that a sample size of 16 lesions per intervention would detect a difference in the PA improved area between the active treatments and control of 25% (i.e., 60% in treatments groups, 35% for the placebo), assuming a SD of 25, at 95% CI, and two tails, *α* of 0.05 and *β* of 0.8. Therefore, 28 subjects with symmetrical lesions were needed to ensure 48 evaluable lesions, considering a dropout rate of 15%. Permuted block randomization was used to assign treatments on lesions. Statistical analysis was performed using analysis of variance, paired *t* test, *χ* ^2^ test (Fisher if  *n* < 5), and correlation tests with a level of significance set at 5%. PGA was standardized using the kappa test of consistency. Tests were performed using the JMP software 8.0 (Cary, NC, USA). 

## 3. Results

The study recruited 28 children affected by PA, with 19 lesions to evaluate in the calcitriol group, 18 in the tacrolimus group, and 19 in the placebo group. The median age was 9 years (range 3–17 years). The demographics, mean duration of PA, mean affected area, and measure values are shown in Table 1.

**Figure 1:**
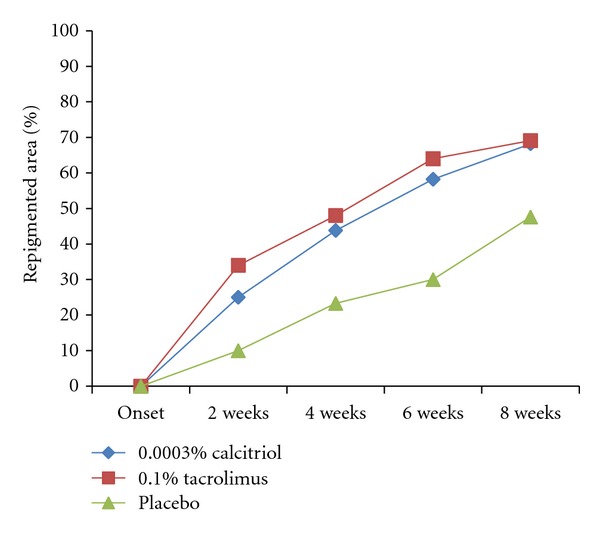
Mean percentage change in the depigmented area of PA target lesions during the study for calcitriol (*n* = 19), tacrolimus (*n* = 18), and placebo (*n* = 19). At 8 weeks, there were differences among groups (one-way ANOVA, *P* < 0.001), but no difference was noted between calcitriol and tacrolimus (*t*  test, *P* = 0.9).

Reductions from baseline in PA lesions in the calcitriol and tacrolimus groups, expressed as mean ± SD percentage change, were significantly higher than those obtained by placebo from week 4 to week 8 (*P* ≤ 0.001). At the end of the study, PA lesions were significantly reduced in the calcitriol and tacrolimus groups compared with the placebo group, as demonstrated by the  mean ± SD percentage change from baseline lesion: 68.2 ± 24.7%, 69.1 ± 25.1%, and 47.6 ± 28.9%, respectively (Figure 1). This data showed no statistical significance in the PA improved area for both drugs (*P* = 0.9).

**Figure 2:**
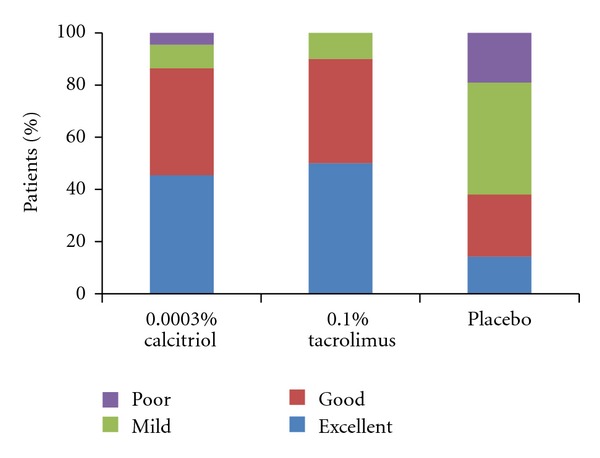
Physician's assessment of response at PA target lesions at the end of treatment (week 8). Calcitriol and tacrolimus efficacy was rated equally for good and excellent response (*P* = 0.6, and *P* = 0.8, *χ* ^2^ test). Placebo response was significantly lower compared to both drugs in the former categories (*P* < 0.001, *χ* ^2^ test).

**Figure 3:**
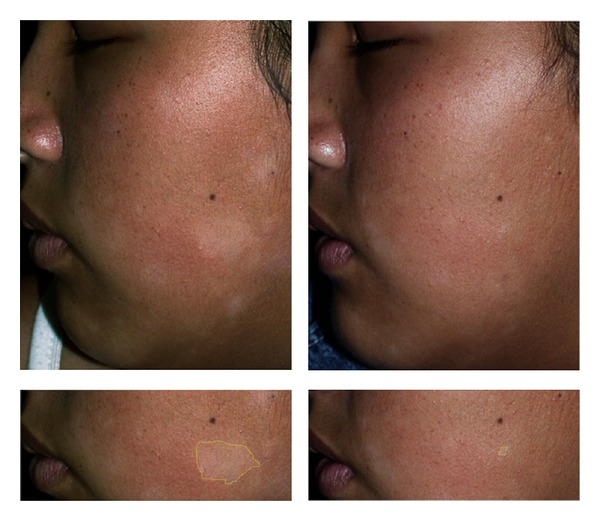
PA lesion treated with 0.0003 % calcitriol in a 10-year-old girl, view at onset (left) and 8 weeks later (right). In the lower picture, target lesion was outlined to measure the affected area showing the excellent clinical response.

**Figure 4:**
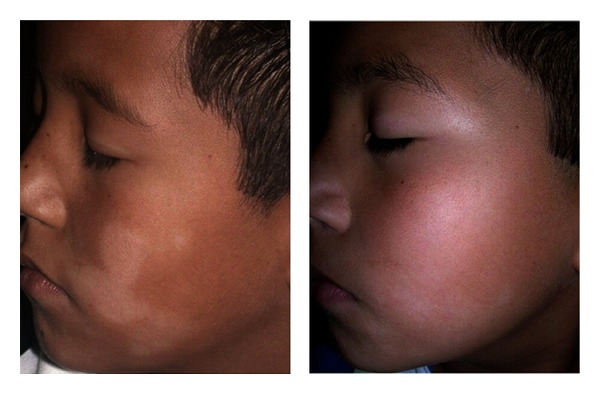
PA lesion treated with 0.1% tacrolimus ointment in a 7-year-old boy: onset and 8 weeks later with excellent improvement.

With respect to the PGA score at the target lesions, 45.4%, 50% and 14.2% of the patients treated with calcitriol, tacrolimus or placebo received a rating of excellent response at week 8 (Figure 2). Altogether 40.9%, 40% and 23.8% of patients in the respective interventions rated their lesions as good. Therefore, there was a range of response from less than 20% to more than 90% in the three groups. Figures 3, and 4 show lesions with excellent improvement by calcitriol and tacrolimus. 

At the end of the study, the average colorimetric changes evaluated by the L*Δ showed significant repigmentation in the groups of lesions treated with calcitriol and tacrolimus (*P* = 0.01, and *P* = 0.001 resp.). There was no difference in the group of lesions treated with placebo (*P* = 0.09), (Table 2). In spite of these differences, we observed a notorious relationship between the digitally measured percentage of improved area, and the reduction in the L*Δ values for calcitriol, tacrolimus and placebo. The correlation coefficients were *r* ^2^ = 0.71 (*P* < 0.001), *r* ^2^ = 0.79 (*P* < 0.001), *r* ^2^ = 0.79 (*P* < 0.001), respectively.

PA lesions exhibited TEWL levels 30% approximately higher than perilesional skin at the beginning of the study. The average TEWL value in unaffected skin was 11.2 ± 3.2 g/m^2^/h, compared to PA values of 14.9 ± 5 g/m^2^/h, 14.8 + 5.6 g/m^2^/h, and 15.1 ± 4.8 in the calcitriol, tacrolimus and placebo groups (*P* = 0.001). At 8 weeks, the mean TEWL changes (TEWLΔ), denoted a significant reduction in the lesions treated with calcitriol and tacrolimus (*P* = 0.008, and *P* = 0.01 correspondingly), but not in those treated with placebo (*P* = 0.63). These data represented a mean reduction of 67%, 57%, and 12.5% respectively in the TEWL for calcitriol, tacrolimus and placebo.

**Figure 5:**
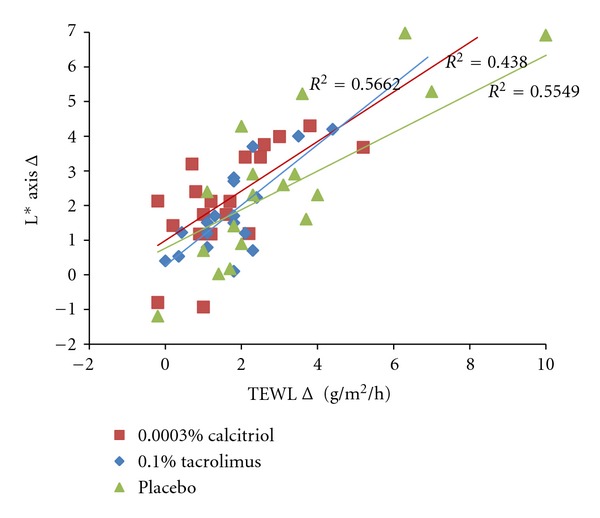
Relationship and regression line between the repigmentation of the PA affected area, represented by the luminosity change (L*Δ), and the corneal stratum integrity change represented by the TEWL Δ for the three interventions. The relationship was significative (*P* < 0.001) for all of them.

We found a significative relationship between repigmentation and the reduction of TEWL in PA-treated lesions. This was analysed by means of the values of TEWLΔ and L*Δ for the calcitriol, tacrolimus and placebo-treated lesions. The correlation coefficients were *r* ^2^ = 0.43 (*P* = 0.002), *r* ^2^ = 0.56, (*P* = 0.003), and *r* ^2^ = 0.54 (*P* = 0.004), respectively (Figure 5).

Concerning side effects, there was a transient complain of burning sensation in 4 patients in the tacrolimus-treated lesions. However, we did not observe visual erythema, neither did we observe significant changes in a* colorimetric values (a*Δ) on PA plaques, at baseline, during, or at the end of the study for any of the interventions (Table 2).

## 4. Discussion

This is the first study to describe the efficacy of calcitriol compared with tacrolimus or petrolatum as placebo for the treatment of endemic PA in children patients. The study showed that after 8 weeks of treatment the clinical efficacy of calcitriol was comparable to that of tacrolimus. The efficacy of petrolatum was also noticed, but was lower compared to these active drugs.

It has been reported that tacrolimus is an effective treatment for atopic-related PA [11]. Therefore, current literature suggests that PA etiology could be mainly inflammatory [2, 9]. However, in our study the plaques of PA did not show elevated redness colorimetric values (a*) that could involve major inflammation, but we found a significantly impaired permeability barrier in PA. TEWL as a marker of barrier function was increased in all the PA lesions compared to non-apparent affected skin. Interestingly, lesions that responded to the interventions showing a pigmented increase simultaneously showed an important reduction in the TEWL. This suggests that PA may have a local skin barrier defect that may be associated to sebaceous glands atrophy and lipids deficiency, which may contribute to the long duration and frequent relapses [3, 4]. 

The improvement observed with calcitriol and tacrolimus in PA patients could be explained by their regulatory role in the melanocyte development and melanogenesis [18, 21, 22] and by an induced restoration of functional defects of barrier due to intrinsic drug properties, in addition to the protective effect of the ointment vehicle mainly composed by petrolatum, and not exclusively to their anti-inflammatory properties. Besides these hallmarks and its cell-proliferative regulation properties [14–17], topical calcitriol is able to restore the epidermal permeability and antimicrobial barrier via activation of the cutaneous vitamin D pathway and also to increase epidermal and antimicrobial peptides [23]. These pharmacological features could explain the improvement seen in the calcitriol treated side.

Another significant fact is that we neither indicated sunscreen use, nor influenced sun exposure habits but we observed notorious improvement; this suggests that most important factors other than UV radiation might be involved in the pathogenesis of this condition. These results made us wonder if steroids or immunosuppressors are justified for this disease, besides considering its reported adverse effects (i.e., cutaneous atrophy, carcinogenic risk, or infections) [24, 25].

Although PA has a chronic course, it has not been extensively studied due to its self-limited and not so grave health implications. Therefore, we have a lack of controlled studies with limited knowledge about its therapy and pathogenesis. Based on the results of our study, we could suggest that calcitriol is at least as effective as tacrolimus, and superior to petrolatum in endemic PA, offering some clinical advantages to the pediatric population. Side effects such as skin burning sensation and immunosupression would be absent compared to tacrolimus, besides the potential of avoiding carcinogenic risk.

These findings matched with the expectations exposed in the theoretical frame, but require confirmation in larger, longer-term studies.

## Figures and Tables

**Table 1:** Demographics, and clinical features of the 28 patients at baseline.

Age (years), mean (SD)	10 (3.6)
Gender, *n* (%)	
Male	15 (54)
Female	13 (46)

Phototype, *n* (%)	
IV	7 (25)
V	21 (75)

Duration of PA (months)	
Mean (SD)	9.6 (10.6)

Previous treatments, *n* (%)	10 (38)

Target lesions	
Area (cm^2^), mean (SD)	3.9 (1.3)
L^**∗**^ axis, mean (SD)	56.7 (2.7)
a^**∗**^ axis, mean (SD)	11.7 (1.5)
TEWL (g/m^2^/h), mean (SD)	14.9 (5)

**Table 2:** Changes in repigmented area (%), colorimetric values (L*, a*), and TEWL (g/m^2^/h) on target lesions of PA. Data are shown initially, and at the end of study for calcitriol, tacrolimus and placebo.

	0.0003% Calcitriol (*n* = 19)			0.1% Tacrolimus (*n* = 18)			Placebo (*n* = 19)		
	Onset	8 weeks	*P*	Onset	8 weeks	*P*	Onset	8 weeks	*P*
Reduced area (%)	0	68.2 (24.7)	<0.001	0	69.1 (25.1)	<0.001	0	47.6 (28.9)	<0.001
L*Δ	4.2 (4.3)	2.3 (1.9)	0.01	4.1 (1.1)	1.8 (1.2)	0.001	4.3 (2.8)	2.7 (2.2)	0.09
a* Δ	−0.4 (1.4)	0.1 (2.1)	0.35	−0.5 (1.5)	−0.3 (1.1)	0.65	0.4 (1.1)	−0.2 (1.6)	0.09
TEWL Δ	4.8 (4.3)	1.6 (1.3)	0.008	4 (3.7)	1.7 (1)	0.01	4 (4.1)	3.5 (3)	0.63

Δ = lesional − perilesional values.

Data are expressed as mean, standard deviation in parenthesis.
